# Phylogenetic informativeness analyses to clarify past diversification processes in Cucurbitaceae

**DOI:** 10.1038/s41598-019-57249-2

**Published:** 2020-01-16

**Authors:** Sidonie Bellot, Thomas C. Mitchell, Hanno Schaefer

**Affiliations:** 1 Royal Botanic Gardens, Kew, TW9 3DS Richmond, UK; 2 Plant Biodiversity Research, Department Ecology & Ecosystem Management, Technical University of Munich, Emil-Ramann Strasse 2, 85354 Freising, Germany

**Keywords:** Phylogenetics, Speciation

## Abstract

Phylogenomic studies have so far mostly relied on genome skimming or target sequence capture, which suffer from representation bias and can fail to resolve relationships even with hundreds of loci. Here, we explored the potential of phylogenetic informativeness and tree confidence analyses to interpret phylogenomic datasets. We studied Cucurbitaceae because their small genome size allows cost-efficient genome skimming, and many relationships in the family remain controversial, preventing inferences on the evolution of characters such as sexual system or floral morphology. Genome skimming and PCR allowed us to retrieve the plastome, 57 single copy nuclear genes, and the nuclear ribosomal ITS from 29 species representing all but one tribe of Cucurbitaceae. Node support analyses revealed few inter-locus conflicts but a pervasive lack of phylogenetic signal among plastid loci, suggesting a fast divergence of Cucurbitaceae tribes. Data filtering based on phylogenetic informativeness and risk of homoplasy clarified tribe-level relationships, which support two independent evolutions of fringed petals in the family. Our study illustrates how formal analysis of phylogenomic data can increase our understanding of past diversification processes. Our data and results will facilitate the design of well-sampled phylogenomic studies in Cucurbitaceae and related families.

## Introduction

Rapid advances in next generation sequencing techniques continue to make it easier and more affordable to produce large amounts of sequence data from fresh material as well as from old herbarium collections^1^. A popular approach is the sequencing of the whole chloroplast genome, with currently more than 2,800 plastomes available^2^, and its analysis is improving our understanding of the tree of life^3^. While plastome sequences are easy to obtain and can result in well-resolved phylogenies, incongruence with signal from the nuclear genome is common^4^ and the number of informative sites in the relatively conserved angiosperm plastome is much lower than in the nuclear genome^5^. Nuclear loci, however, are more difficult to sequence at low cost, and their higher substitution rate makes them more easily subject to homoplasy. Target sequence capture approaches are an efficient way to reduce the cost of sequencing nuclear loci by increasing their representation in the DNA to be sequenced^6^. The selection of loci to target depends on the research question, but often requires a trade-off between maximizing phylogenetic signal and minimizing the risk of homoplasy. A similar trade-off must be achieved when selecting what loci to use for phylogenetic analyses among hundreds of previously sequenced loci, regardless if they were obtained by targeted or whole genome sequencing. Finally, classifying loci according to their phylogenetic signal can provide a basis to interpret polytomies in taxon phylogenies and conflicts between locus trees. Combining methods that detect such conflicts and methods that characterize locus information content will therefore be instrumental to make the most of next generation sequencing for investigating diversification events across the tree of life^7^.

The development of methods to identify conflicts between loci or nucleotide sites in phylogenomic datasets is an active area of research^8^, and some of these methods can assess the amount of signal underlying conflicts^9^. This allows to distinguish between conflicts due to lack of phylogenetic signal and conflicts due to biological phenomena such as horizontal gene transfer (HGT), incomplete lineage sorting (ILS), hybridization or homoplasy^9^. In the case of lack of signal, estimating the probability of resolution of a polytomy could help deciding what additional sequencing effort (if any) is likely to provide resolution^10^. Methods to estimate probability of resolution still have to be refined^11^ but they ultimately could allow to formalize claims of “hard” polytomies. On the other hand, when the conflicts are supported by phylogenetic signal, knowing how likely the signal is to be homoplasious allows to distinguish conflicts due to homoplasy from those due to other events (such as hybridization, ILS or HGT) that may be of higher relevance to understand taxon diversification. Different metrics of phylogenetic signal have been proposed^12^, some of them allowing to differentiate between signal and noise (homoplasy)^13^. One of these methods uses site rate estimates to profile locus phylogenetic informativeness (PI) throughout a given epoch^14,15^. Locus PI can be integrated over different epochs of a group’s history, allowing to determine for which epoch is a locus most informative, and then for which epochs it may be uninformative (younger epochs) or homoplasious (older epochs). In addition, the PI of all loci for a given epoch can be compared to identify the set of loci that are most likely to be useful to solve a given polytomy. These properties of PI profiles can be used to select loci that will improve phylogenetic resolution or to provide interpretations for the lack of it. The latter still requires theoretical developments and empirical tests^11,12^, but the former should already be applicable to real-world phylogenetic challenges.

The cucurbit family is an excellent candidate to test the informativeness of the plastome in comparison to nuclear regions and to explore the potential of phylogenetic informativeness analyses. In Cucurbitaceae, a mostly tropical plant family with about 1000 species^16^, complete plastomes have so far only been published for around 30 species, mainly medicinal plants or crop species and their closest relatives^17,18^. Due to the large number of crop species in the family, the group has been well classified in the past decades both morphologically^19,20^ and through the analysis of a small set of chloroplast (*rbcL*, *matK*, *trnL*, *rpl20*-*rps12*) and nuclear (ITS) DNA regions, the latter for more than 60% of the cucurbit species worldwide^21–23^. In combination, these data have resulted in a reasonably resolved phylogeny estimate for the family, which is largely compatible with biogeographical data^16^, but several key relationships remain unresolved so far. For example, the relationships in the tribe Sicyeae, a group with several pollinator shifts and changes in diversification rate, probably linked to the evolution of fringed petals^24^, are still poorly resolved. In particular, the morphologically and geographically well-characterised snake gourds, *Trichosanthes*, with c. 90 Asian species and very special pollination biology^25^ are frequently recovered as paraphyletic^22,23^ or monophyletic with low bootstrap support (BS)^26^. The position of several early branching cucurbits, such as the floral oil-producing *Indofevillea* and *Siraitia* also remains uncertain^22^. These uncertainties hamper evolutionary and biogeographical studies of the cucurbit family including ancestral floral trait inference and the analysis of sexual system evolution.

Here, we present the first phylogenomic study of Cucurbitaceae, where a genome skimming approach was used to produce full plastomes plus a set of single-copy nuclear genes for all but one of the 15 cucurbit tribes, with increased sampling in Sicyeae. We demonstrate the potential of genome skimming data and phylogenetic informativeness methods to resolve divergence events at different epochs of the Cucurbitaceae history and provide new insights on their diversification.

## Results

### Plastome structure and gene content are generally conserved across Cucurbitaceae

**Figure 1:**
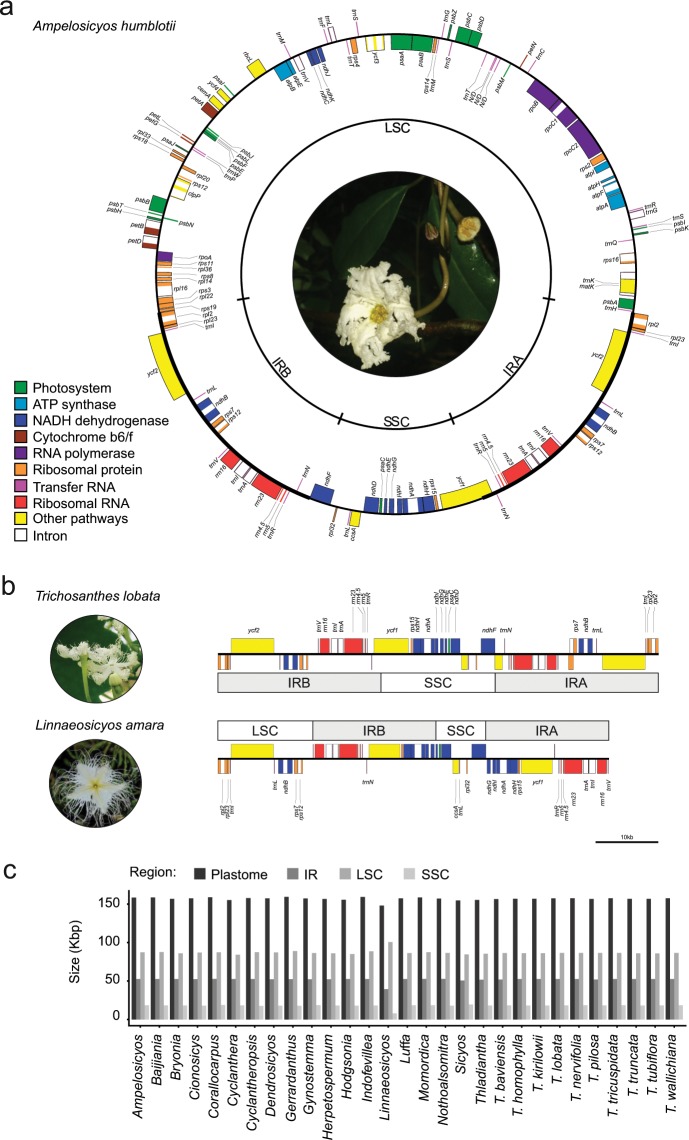
Structure and gene content of Cucurbitaceae plastomes. (**a**) Plastid genome of *Ampelosicyos humblotii* (Picture: HS). (**b**) Comparison of the location and gene content of the inverted repeat and small single copy regions of *Trichosanthes lobata* and *Linnaeosicyos amara* (Pictures: HS and TM). The large single copy region of *T. lobata* and most of that of *L. amara* were truncated to improve visualisation. (**c**) Size comparison of the different plastid genome regions across Cucurbitaceae; the sizes of both copies of the inverted repeat were summed.

The plastid genomes of all 29 Cucurbitaceae species were structurally identical so we show only the plastome of *Ampelosicyos humblotii* in Fig. 1a as an example. Each plastome could be assembled in a circular molecule consisting of a large and a small single-copy (LSC and SSC) region separated by an inverted repeat (IR). Gene content and order was identical in all species, with 79 protein-coding genes (of which 14 had introns), 30 tRNAs, (of which six had introns) and four ribosomal RNAs, when counting only once the loci located in the IR region (Fig. 1a). A comparison of the IR boundaries in *Linnaeosicyos amara* and *Trichosanthes lobata* is presented in Fig. 1b. In all species the IR started with the gene *rpl2* and ended inside the gene *ycf1*, except for *L. amara*, where it started before *trnV*-GAC and ended in *ndhG*, so that part of what was the SSC in other Cucurbitaceae was in the IR in *L. amara* (Fig. 1b). The bar plots of Fig. 1c represent the size distribution of the different plastome regions across species. Most plastomes are between 154,564 and 159,232 bp long, with an IR region between 25,328 and 26,340 bp long, an LSC region between 84,165 and 88,912 bp long and an SSC region between 17,587 and 19,486 bp long. The only outlier was the plastome of *L. amara*, which, due to its IR boundaries switch (Fig. 1b) is only 147,874 bp long and has a longer LSC (100,495 bp) and shorter IR (19,688 bp) and SSC (8,003 bp) regions than the other species (Fig. 1c).

### Large plastid and nuclear datasets failed to resolve relationships between Cucurbitaceae tribes

**Figure 2:**
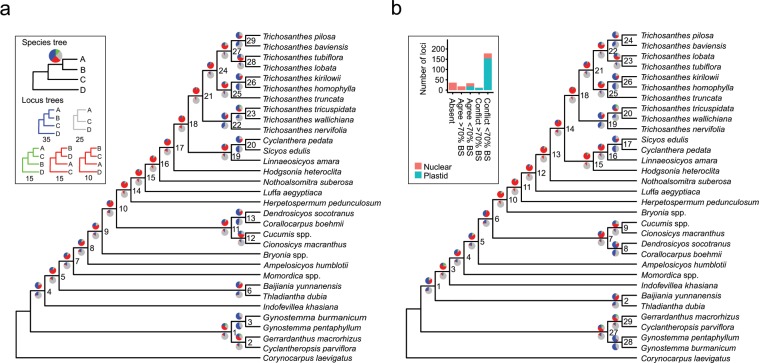
Phylogenies of Cucurbitaceae based on all plastid and nuclear loci. (**a**) RAxML maximum likelihood (ML) tree inferred from a concatenated alignment of all loci. Inset: Visual explanation of how locus tree support for a given species tree clade can be represented as a pie chart; number of loci yielding the same topology are indicated under each locus topology. The pie chart then represents the percentage of loci that agree (blue), disagree (one of the main alternatives: green, other alternatives: red), or are neutral (grey) with respect to the clade. (**b**) ASTRAL multispecies coalescent phylogeny inferred by summarizing all nuclear locus trees and a single plastid tree obtained by ML analysis of a concatenated alignment of all plastid loci. Inset: bar plot representing locus support for node 25. See Results section 2 for details. Pie charts above branches represent locus support for the clade descending from the branch. Pie charts below branches represent the same but after collapsing nodes with BS < 70% in locus trees. Node labels are arbitrary numbers for easier description in the text.

The analysis of all plastid loci and of 58 nuclear loci obtained from the genome skimming data or by PCR (see Methods) resulted in different phylogenetic hypotheses for Cucurbitaceae tribes and *Trichosanthes* species, presented in Fig. 2 and Supplementary Fig. S1. These trees were obtained by analysing a concatenated alignment of all plastid and nuclear loci with RAxML^27^ (hereafter RAxML tree; Fig. 2a) or by summarizing locus trees using ASTRAL-III^28^, either after concatenating all plastid loci (hereafter ASTRAL-conP; Fig. 2b), or by keeping all loci separate (hereafter ASTRAL-sepP; Suppl. Fig. S1a). The topologies of the ASTRAL-sepP and RAxML trees were identical except for the position of *Luffa*, which nested among other Sicyeae in the former (Suppl. Fig. S1a) but grouped as sister to other Sicyeae in the latter (Fig. 2a) and the ASTRAL-conP tree (Fig. 2b). This topological conflict is reported in Table 1, in addition to the three other topological conflicts we recovered, concerning the positions of *Hodgsonia*, *Bryonia*, and *Indofevillea*. In each case, the conflict was between the ASTRAL-conP tree (Fig. 2b) and the two other trees (Fig. 2a and Suppl. Fig. S1a). Branch lengths and bootstrap support are presented in Supplementary Fig. S1b for the RAxML species tree. All nodes had high (≥70%) to maximal BS, including the nodes that corresponded to, or surrounded, the branching points of the four conflicting taxa (Table 1, Suppl. Fig. S1b). Despite their high BS, these nodes were always preceded by short branches, suggesting that low amounts of phylogenetic signal may be responsible for the conflicts.

**Table 1:** Conflicts between species trees. Letters indicate a topological alternative, and numbers in brackets refer to the corresponding nodes on Figs. 2a,b and Suppl. Fig. S1a.

Lineage	RAxML (Fig. 2a)	ASTRAL-conP (Fig. 2b)	ASTRAL-sepP (Suppl. Fig. S1a)
*Luffa*	A (16)	A (13)	B (13)
*Hodgsonia*	A (18)	B (15)	A (15)
*Bryonia*	A (10)	B (10)	A (7)
*Indofevillea*	A (5)	B (3)	A (2)

Percentages of locus trees agreeing or conflicting with each species tree clade are represented by pie charts on the species trees. Pie charts above branches were obtained from fully bifurcating locus trees, while these below branches were obtained from locus trees where nodes with low BS (<70%) had been collapsed. The percentage of locus trees agreeing with the clade is shown in blue. The percentages of locus trees conflicting with the clade are shown in green (one of the most represented alternative topologies) and red (all other alternatives). The grey corresponds to the percentage of locus trees that neither agreed nor conflicted with the species tree topology, either because they lacked the relevant taxa, or because collapsing their nodes with low BS resulted in a polytomy (inset on Fig. 2a). For all clades of all species trees, the percentages of agreeing + conflicting loci decreased from up to 100% (e.g. node 21 on Fig. 2a) to up to 60% (e.g. node 3 on Fig. 2a) after collapsing nodes with low support in the locus trees, confirming that many loci had low phylogenetic signal. For many clades, collapsing nodes with low support in the locus trees resulted in increased support for the species tree clade compared to the alternatives, suggesting that most lineages had only few well-supported intra-genomic conflicts and that these conflicts were insufficient to blur species history. For the nodes corresponding to the branching points of the conflicting taxa (*Bryonia*, *Luffa*, *Hodgsonia*, and *Indofevillea*; Table 1), collapsing nodes with low support in the locus trees resulted in evenly low (<5%) locus tree support for the species tree clades and for their alternatives. This was also the case for nodes around these branching points, such as nodes 14, 15, and 21 on Fig. 2a and nodes 11, 12 and 18 on Fig. 2b, which involved *Herpetospermum* and the ancestor of *Trichosanthes*. In these cases, the few intra-genomic conflicts were therefore enough to blur the phylogenetic signal. Loci were also classified by genomic compartment to detect signal for nucleo-cytoplasmic conflicts. Only one possible case could be found, where the placement of *T. truncata* as sister to *T. kirilowii + T. homophylla* was highly supported only by nuclear loci and highly conflicted by plastid loci and by only one nuclear locus (inset on Fig. 2b).

### Loci could be classified according to their potential utility across the phylogeny

**Figure 3:**
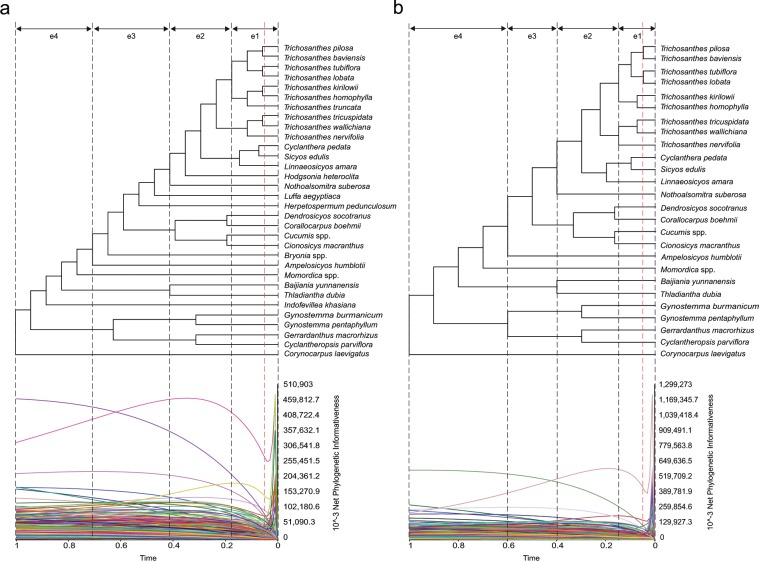
Phylogenetic informativeness of plastid and nuclear loci across the history of Cucurbitaceae. (**a**) Same ML phylogeny of Cucurbitaceae as in Fig. 2a and net phylogenetic informativeness of each locus. (**b**) Same as in (a) but after excluding conflicting taxa. Each coloured line represents a single locus. Labels e1, e2, e3, and e4 refer to the epochs in which we partitioned the history of Cucurbitaceae. See Methods section 4 for details about conflicting taxa and epochs.

To distinguish between non-informative, informative and potentially misleading loci, we estimated their phylogenetic informativeness (PI) at four epochs of the Cucurbitaceae history (see Methods section 4). Figures 3a,b display the PI profiles of all loci across the four epochs, depending on inclusion or exclusion of conflicting taxa. Some loci (18 in the tree including all taxa and 31 in the tree without conflicting taxa) showed very high rate peaks before time t = 0.05 (red line on Fig. 3). These peaks are artefacts which occur when the function used to estimate substitution rates is unable to give precise estimates for sites with indels or ambiguities (http://phydesign.townsend.yale.edu/faq.html#phantomPeak). In a conservative approach, we discarded these loci from further PI calculations, and PI was only integrated from t = 0.05 onwards for the other loci. For each epoch e1 to e4, an integrated PI (iPI) was estimated for each locus by integrating the locus PI over the epoch^15^. The signal accumulated during an epoch is likely to be blurred by signal accumulated at more recent epochs, so if a locus maximal iPI is in a more recent epoch than the epoch considered, the locus could be misleading for the epoch. We therefore penalized our estimates of iPI for each locus and each epoch if the maximal locus iPI was in a more recent epoch. This allowed us to estimate the risk of a locus to be misleading by considering the difference between its iPI and its penalized iPI (iPI_pen_) for a given epoch (see Methods section 4 for how iPI_pen_ was calculated). Integrated PI values and their penalized versions are reported on Supplementary Fig. S2 for each locus and each epoch. Regardless if conflicting taxa were removed (Suppl. Fig. S2b) or not (Suppl. Fig. S2a), the iPI_pen_ of all loci were very similar to their iPI for e1 and e2, showing a low risk of homoplasy. For e3, iPI_pen_ was inferior to iPI for some nuclear loci, but this effect disappeared when conflicting taxa were removed. For e4, many nuclear genes had iPI_pen_ < iPI, and this was accentuated when problematic taxa were removed (Suppl. Fig. S2b). The two contrary patterns observed for e3 and e4 illustrate the unpredictable influence that problematic taxa can have on PI profiles. The high difference observed between iPI_pen_ and iPI for some loci for e3 and/or e4 suggested a high homoplasy potential for these loci, thus warranting their utilisation to resolve divergences that occurred in these epochs. These loci often had a higher iPI than the other loci for e1 and e2, suggesting that they may be useful to resolve divergences that occurred in these more recent epochs.

**Figure 4:**
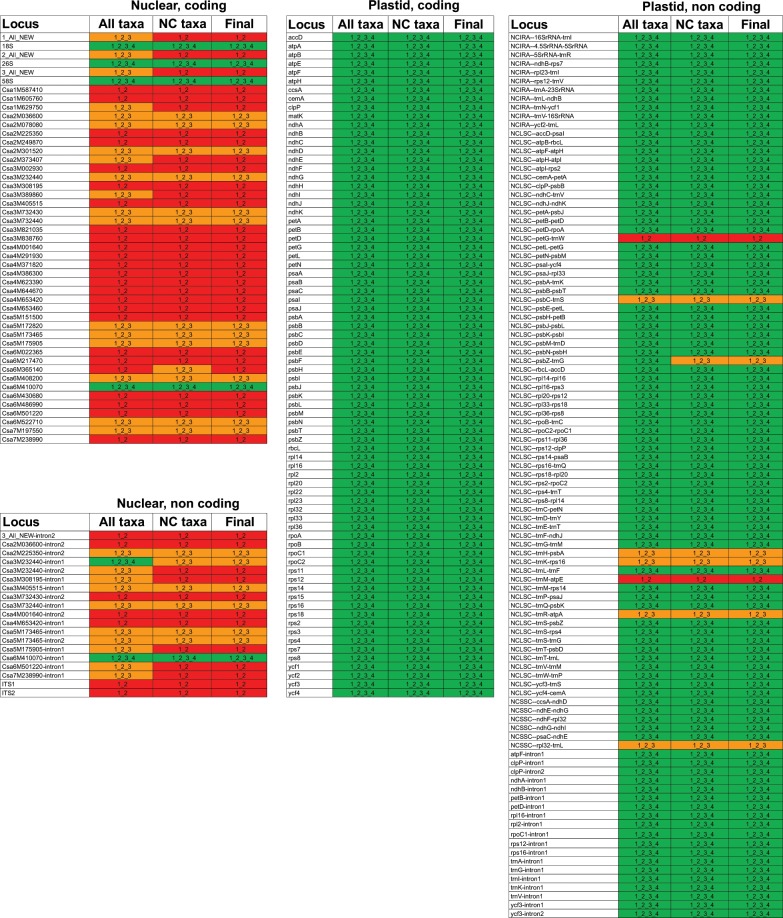
Predicted misleading signal in plastid and nuclear loci for inference of Cucurbitaceae relationships in different epochs. Loci classified as “1-2-3-4” (green) were predicted to be non-misleading in all epochs, loci classified as “1-2-3” (orange) were predicted to be misleading for divergence events that happened in epoch 4, and loci classified as “1-2” (red) were predicted to be misleading for divergence events that happened in epochs 3 or 4. NC: non conflicting. The “Final” column provides our final classification of each locus, corresponding to the most misleading class among these in columns “All taxa” and “NC taxa”.

For each epoch, we classified loci as misleading if iPI_pen_ < iPI for that epoch and as non-misleading otherwise. Figure 4 summarises this classification, depending on inclusion or exclusion of conflicting taxa, which mostly did not change the classification. Most plastid loci were classified as non-misleading even for old divergences (“1-2-3-4”, green on Fig. 4), regardless if they were protein coding genes, introns or intergenic spacers. Eight plastid loci were classified as non-misleading for epochs e1, e2 and e3 but misleading for e4 (“1-2-3”, orange on Fig. 4), namely *psbC–trnS*, *psbZ–trnG*, *trnH–psbA*, *trnK–rps16*, *trnR–atpA*, and *rpl32–trnL*. Only the two plastid intergenic spacers *trnM–atpE* and *petG–trnW* were classified as non-misleading for epochs e1 and e2 but misleading for e3 and e4 (“1-2”, red on Fig. 4). On the contrary, most nuclear loci were classified as non-misleading for epochs e1 and e2 but misleading for e3 and/or e4. Only the coding sequence and the intron of gene Csa6M410070, and the ribosomal DNAs 26 S, 18 S and 5.8 S were classified as non-misleading (“1-2-3-4”, green on Fig. 4) for e3 and e4.

Integrated PI was plotted according to utility and epoch on Supplementary Fig. S3, to assess how phylogenetically informative were the loci classified as non-misleading. We observed a trend for loci that were non-misleading in all epochs (“1-2-3-4”) to have the lowest iPI. However even among these loci some had an iPI similar to the average iPI of loci that were only non-misleading for the most recent epochs (“1-2” and “1-2-3”). This trend was conserved regardless if we included conflicting taxa (Suppl. Fig. S3a) or not (Suppl. Fig. S3b).

### Data filtering clarified relationships in Cucurbitaceae despite general lack of signal

**Figure 5:**
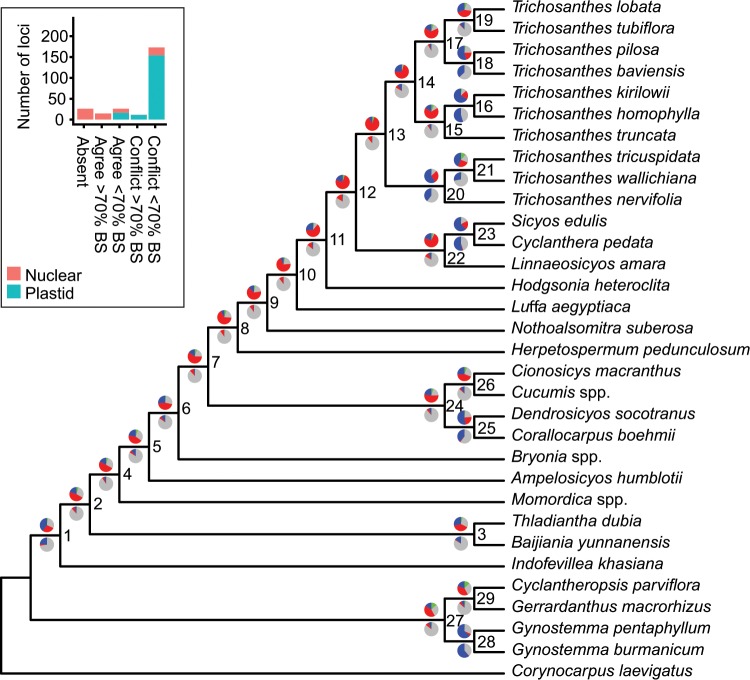
ML tree inferred from a concatenated alignment of all plastid and nuclear loci after data filtering. Pie charts above branches represent the percentage of loci that agree (blue), disagree (one of the main alternatives: green, other alternatives: red), or are neutral (grey) with respect to the clade descending from the branch. Pie charts below branches represent the same but after collapsing nodes with BS < 70% in locus trees. Node labels are arbitrary numbers for easier description in the text. Inset: bar plot representing locus support for node 15. See Results section 2 and Methods section 4 for details.

To decrease the impact of homoplasy on our phylogenetic inferences, we removed taxa from locus alignments if the locus was considered misleading for the epoch in which the taxon diverged from its sister taxon. Figure 5 shows the RAxML tree obtained after such data filtering. Pie charts follow the same legend as in Fig. 2 (see Results section 2). The ASTRAL trees obtained after data filtering either when keeping plastid loci separate (ASTRAL-sepP) or when concatenating them (ASTRAL-conP) are presented in Supplementary Fig. S4a,b respectively. The four lineages that prior to data filtering changed position depending on the analysis became stable across analyses after data filtering (Table 2). These placements followed the ones recovered before data filtering by the ASTRAL-sepP analysis (Fig. 5 and Suppl. Fig. S1a).

**Table 2:** Conflicts between species trees. Letters indicate a topological alternative, and numbers in brackets refer to the corresponding nodes on Figs. 2a,b and 5, and Suppl. Fig. S1a, S4a,b.

Lineage	Before data filtering			After data filtering		
	RAxML (Fig. 2a)	ASTRAL-conP (Fig. 2b)	ASTRAL-sepP (Suppl. Fig. S1a)	RAxML (Fig. 5)	ASTRAL-conP (Suppl. Fig. 4b)	ASTRAL-sepP (Suppl. Fig. 4a)
*Luffa*	A (16)	A (13)	B (13)	B (10)	B (13)	B (13)
*Hodgsonia*	A (18)	B (15)	A (15)	A (12)	A (15)	A (15)
*Bryonia*	A (10)	B (10)	A (7)	A (7)	A (7)	A (7)
*Indofevillea*	A (5)	B (3)	A (2)	A (2)	A (2)	A (2)
*T. truncata*	A (25)	A (25)	A (22)	A (15)	A (25)	B (22)
*T. tricuspidata* + *T. wallichiana* + *T. nervifolia*	A (21)	A (18)	A (18)	A (13)	B (16)	A (18)

Comparing the pie charts on Fig. 5 with these on Supplementary Fig. S1a revealed that, after data filtering; (i) support for the placements of *Luffa* and *Hodgsonia* did not change, (ii) a lower percentage of loci agreed with the placement of *Bryonia*, but the percentage of loci supporting the most represented alternative did not change and remained lower, and (iii) the percentage of loci supporting the most represented alternative to the placement of *Indofevillea* decreased and became lower than the percentage of loci in agreement. The other nodes that did not have a high percentage of loci in agreement before data filtering (nodes 14 and 15 on Fig. 2a involving *Herpetospermum* and node 21 on Fig. 2a, involving the most recent common ancestor of *Trichosanthes*) were more highly supported after data filtering, either because their percentage of loci supporting the most represented alternative decreased (node 8 on Fig. 5) or because their percentage of loci in agreement increased (nodes 9 and 13 on Fig. 5).

Although data filtering clarified the above-described relationships, it also perturbated the resolution of two nodes that were stable before, as summarised in Table 2. The position of *T. truncata* as sister to *T. kirilowii* + *T. homophylla* was recovered after data filtering in the RAxML analysis (Fig. 5) and in the ASTRAL-conP analysis (Suppl. Fig. S4b), but not in the ASTRAL-sepP analysis (Suppl. Fig. S4a), where it was instead placed as sister to all *Trichosanthes* except the clade *T. nervifolia* + *T. wallichiana* + *T. tricuspidata*. This conflicting position was, however, less well supported, with a higher percentage of loci supporting the most represented alternative than agreeing with the placement. Bar plots in the insets of Fig. 5 and Suppl. Fig. S4a showed that the cause of this disagreement was unlikely to be a conflict between nuclear and plastid evolutionary histories because for both alternative placements of *T. truncata*, only nuclear loci agreed and both plastid and nuclear loci disagreed. Finally, the position of the clade *T. nervifolia* + *T. wallichiana* + *T. tricuspidata* as sister to other *Trichosanthes* was recovered after data filtering in the RAxML analysis (Fig. 5) and in the ASTRAL-sepP analysis (Suppl. Fig. S4a), but not in the ASTRAL-conP analysis (Suppl. Fig. S4b), where it was instead placed as sister to clade *Sicyos* + *Cyclanthera* + *Linnaeosicyos*. This conflicting position was, however, again less well supported, with a higher percentage of loci supporting the most represented alternative than agreeing with the placement.

## Discussion

This study showed that, despite having diverged up to 60 million years ago^22^, the plastomes of Cucurbitaceae are highly conserved in size, structure, gene content, and gene order. Such conservation is not unusual in angiosperms^29^, and may be partly due to the essential role played by plastomes in the photosynthesis pathway, since non-photosynthetic plants are notably more variable in structure and gene content^30^. Selection pressures other than the need to perform photosynthesis may, however, be involved in the conservation of plastome structure, since families of photosynthetic plants, such as Campanulaceae^31^ or Geraniaceae^32^ show higher structural variation across lower phylogenetic distances. Large-scale studies of full plastomes and life history traits in a well-resolved phylogenetic context are needed to decipher the factors responsible for plastome structure variation in angiosperms. Within Cucurbitaceae, the analysis of more species could provide new insights into plastome evolution if many species-specific variations (such as the different IR of *L. amara*) could be compared.

The difficulties of resolving Cucurbitaceae relationships^22^ have so far impaired our understanding of the highly varied sexual characters and pollination systems of Cucurbitaceae^26,33,34^ with potentially important implications for species conservation and crop breeding. Although higher taxon sampling is required to clarify the phylogeny of Cucurbitaceae, our study shows that sampling more loci can also be beneficial, on the condition that the amount of signal and noise likely to be carried by these loci is carefully evaluated. Evidence is provided for a closer relationship between Fevilleeae and Zanonieae than to Gomphogyneae, and an early divergence of Thladiantheae from the rest of Cucurbitaceae, followed by the divergence of Momordiceae. These relationships were recovered without support by Schaefer and Renner^23^. Additional evidence for the evolution of Sicyeae recovered in the latter study, where *Luffa* was second to diversify from the others after *Nothoalsomithra* and followed by *Hodgsonia* is also provided. In the study of Schaefer *et al*.^22^. *Trichosanthes* was not recovered as monophyletic, and included *Hodgsonia*, as well as a clade formed by *Linnaeosicyos*, *Cyclanthera* and *Sicyos*. Our study provided evidence of the monophyly of *Trichosanthes*. Taken together, our study and the study of De Boer *et al*.^26^, which was focused on Sicyeae and did not provide resolution for the placements of *Hodgsonia* and *Linnaeosicyos*, appear to reveal two independent evolutions of fringed petals in Cucurbitaceae: once in Telfairieae (*Telfairia* and *Ampelosicyos*, incl. *Odosicyos* and *Tricyclandra*), and once in Sicyeae in the common ancestor of *Hodgsonia*, *Linnaeosicyos*, and *Trichosanthes*. The petal fringes were lost in the New World Sicyeae after the divergence of *Linnaeosicyos* and they were also lost in the two *Trichosanthes* lineages that shifted to day flowering. This suggests a high importance of a hawkmoth pollination system which likely existed for more than 30 million years (since the divergences of *Ampelosicyos* and *Telfairia*; *Linnaeosicyos*; *Trichosanthes*)^22^ with only three documented losses^26^.

Phylogenetics are entering an interesting era where outcomes are not only a (set of) species tree(s), but also new insights about the diversification processes that occurred in the group of interest, as we show here with Cucurbitaceae. We found low evidence for strongly supported gene conflicts, suggesting that while incomplete lineage sorting (ILS) may have occurred, it was not a major phenomenon in the oldest epochs of Cucurbitaceae evolution. The occurrence of some degree of ILS in Cucurbitaceae’s past was corroborated by our recovery of different topologies in the ASTRAL and RAxML analyses, which are known to perform differently in the presence of ILS^35^. We could identify one possible case of nuclear–plastid conflict suggesting a past reticulation event with plastid capture, in *T. truncata*. Even though this species is pollinated by generalist hawkmoth species^25^ and could thus have been the result of a hybridisation, the analysis of more genes of more *Trichosanthes* species is required to test this hypothesis. Despite the possible occurrence of ILS and hybridisation events, our results show that difficulties to resolve the backbone of Cucurbitaceae were mostly due to low amounts of phylogenetic signal in plastid loci, and high amounts of noise in nuclear loci for the older epochs. This suggests that the divergence between Cucurbitaceae tribes occurred too rapidly to allow signal accumulation, and/or that too much time has passed to prevent homoplasy blurring this signal. Recent research has revealed a whole genome duplication event in the ancestor of all Cucurbitaceae, which could have contributed to a rapid diversification of the family at that time^36^. Although useful, our classification of loci as misleading or non-misleading based on PI profiles was rough and solely intended to provide a basis for locus selection in this preliminary study. In the future, greater taxon sampling in combination with more sophisticated analyses of signal and noise could improve our inferences, particularly if their utility for pectinate trees is clarified, which is currently a topic of research^11^. We chose not to use locus statistical binning because it has been shown to be misleading when loci do not contain much phylogenetic signal^37^, which was our case. The alternative could be to use site-based rather than locus-based methods^38,39^, but the available methods cannot accommodate large datasets and are not designed for genus-level phylogenies, and/or their robustness to model violations remains to be validated^7^. We therefore refrained from using them until the next phase of Cucurbitaceae phylogenomics, involving a deep sampling at the species level, is reached.

Besides greater taxon sampling, the development of Cucurbitaceae phylogenomics will also require improved selection of loci. We showed that our current set of loci, which was obtained randomly by searching for all single copy genes present in our genome skimming data, contains only limited amounts of phylogenetic signal. One could design a target capture approach to sequence only the most informative/least-misleading loci of our set and use published genomes and transcriptomes of Cucurbitaceae to complete the set with more informative loci. Our search for such loci that could be amplified by PCR (see Methods section 3) revealed hundreds of regions that could potentially be included in a target capture study. Judging from the low variation of many loci used in our study, the same target capture set could probably be used across all Cucurbitales with the possible exception of the holoparasitic Apodanthaceae^40^. Such phylogenomic study would be instrumental to shed light on past radiations, reticulation events, ILS, and character evolution in this order of about 2600 species, including many crop species and the economically important begonias. The selected loci could also be useful for analyses of the closely related Fabales (legume order), Rosales (rose order) and Fagales (oak and beech relatives).

## Methods

### Plant material, DNA extraction and sequencing

At least one representative of each of the 15 Cucurbitaceae tribes except Actinostemmateae was sampled from fresh material collected in Madagascar in March 2013 (*Ampelosicyos*), and in the Dominican Republic in March 2014 and 2015 (*Linnaeosicyos*), as well as from plants cultivated in Freising or from herbarium specimens from E, L, M, P, and TUM. In total, 29 species were newly sequenced, with a special focus on the tribe Sicyeae, which contains the genus *Trichosanthes* (see Supplementary Table S5 for voucher details). Fresh leaf fragments were dried for at least 24 hours in silica gel before grinding in a mixer mill (Retsch MM200, Haan, Germany). Total genomic DNA was extracted using a commercial extraction kit (Nucleospin Plant II kit, Macherey-Nagel, Düren, Germany) following the manufacturer’s protocol.

To gather more phylogenetic signal than could be obtained in previous studies, an approach of genome skimming was undertaken to recover plastid genomes and high copy number nuclear regions (such as the nuclear ribosomal RNAs 18S-5.8S-26S and the ITS1 and ITS2 regions separating them). The total genomic DNA of these species was sent to GENEWIZ (South Plainfield, NJ, USA) for library preparation and multiplexed sequencing on one lane of an Illumina HiSeq 2500 platform. In order to ensure a minimal amount of nuclear data across all taxa, three newly selected (see below) nuclear regions were amplified by PCR in all taxa using the following custom primers (see Methods section 3 for region names): 1_All_NEW: 5′-TATTGCCCCACTCACTCAGC, 5′-TGCCTACACCGTGTAGCATC; 2_All_NEW: 5′-GAAGGTTCACCCACAACCCA, 5′-AGCCAACCTGTGTAGAAGCC; 3_All_NEW: 5′-AATGCTGCTGGGCCATTTTG, 5′-CATCCATCTCCACCAACAAGC and the internal primers 5′-GAGTGGTATTCGTCCTTTGGC, 5′-TCCCTCCAGTAATTGTGACC. Primers were designed with Geneious vs. 8 (Biomatters, Auckland, NZ) and the PCR followed standard protocols for the family^41^. The PCR products were cleaned using ExoSAP (Jena Bioscience, Jena, Germany) and sent to GATC Biotech (Konstanz, Germany) to be sequenced on an ABI Prism 3100-Avant automated sequencer (Applied Biosystems, Foster City, CA, USA).

### Assembly of plastid genomes and nuclear contigs

Illumina sequencing yielded between 9,033,212 and 13,026,096 150 bp-long paired-end reads per sample. Reads were quality checked in the software FASTQC (https://www.bioinformatics.babraham.ac.uk/projects/fastqc/), trimmed of adapters and bases with a phred score < 30 using TrimGalore! (https://www.bioinformatics.babraham.ac.uk/projects/trim_galore/) and de novo assembled with the CLC Genomic workbench v. 7 (https://www.qiagenbioinformatics.com/), resulting in 57,609 to 321,056 contigs per sample. Contigs were aligned (blastn) against a database of 698 plastomes retrieved from GenBank and representing most land plant lineages (list available on demand), the mitochondrial genome of *Citrullus lanatus* (GenBank accession NC014043), and the nuclear genes of *Cucumis sativus* obtained from the CDS dataset of the GenBank project PRJNA80169 v. 2^42^ to classify contigs as belonging to the plastid, mitochondrial or nuclear genomes.

In most cases three or four contigs corresponding to the two single-copy regions and the inverted repeat region of the plastome could be recovered and assembled manually into a circular plastome by checking the reads at the contig borders and choosing the conformation with the highest coverage (knowing that the other conformation would also exist). In a few cases where coverage was too low to make a contig with the CLC assembler, plastome-like contigs were assembled in a circular plastome by iteratively remapping the reads on the contigs (also with CLC) to extend them until they overlapped with other contigs. Mitochondrial and nuclear contigs were not assembled into larger contigs due to insufficient read coverage and/or large repeats preventing unambiguous assembly. Full plastomes were annotated using Geneious v. 8 (Biomatters, Auckland, New Zealand) and *Cucumis sativus* as a reference, and annotations were then manually improved in problematic regions with very small exons or alternative start codons. Plastomes were drawn using OGDraw v. 1.3.1^43^.

### Selection of nuclear genes for phylogenomics in cucurbits

To ensure a minimal amount of nuclear data across all taxa, we looked for a few regions variable across Cucurbitaceae but surrounded by regions conserved enough to allow primer design for PCR amplification and Sanger sequencing. To identify such regions, we modified the protocol of Weitemier *et al*.^6^, originally conceived to design probes for high throughput targeted sequence capture. We used the full genome of *C. sativus* as a reference to infer gene copy numbers and annotations, and we complemented it with the published transcriptomes of *Cucumis sativus*, *Momordica charantia*, *Luffa* sp. and *Siraitia grosvenorii* (Supplementary Table S5) to assess exon variability across Cucurbitaceae. To ensure that the selected loci would contain regions suitable for primer design, CDS containing sections >18 bp matching the *Cucumis* genome with >99% identity were identified using the program BLAT v. 32^44^, and retained if they had homologous sequences with the same highly-conserved regions in the four transcriptomes. CDS showing ≥ 95% total sequence similarity between any of the references were removed using CD-HIT-EST v. 4.5.4^45^, to allow for enough variation across the family. We then discarded CDS that could not be aligned across all references as well as CDS with more than one hit against the reference genome, in order to keep only single-copy genes that could be aligned across the Cucurbitaceae family. Finally, we kept only the 2264 remaining genes that comprised three small exons flanking two introns between 300 and 500 bp long, and the 55 remaining genes that comprised only one exon between 1000 and 1400 bp long. Such properties would allow primer design in exons, and the recovery of the entire region by a single pair of forward and reverse Sanger sequencing reads. We finally arbitrarily chose three test regions among the latter 2319, called in our datasets and figures “1_All_NEW”, “2_All_NEW” and “3_All_NEW”, and corresponding respectively to genes Csa6M497200.1, Csa3M126770.1, and Csa6M406540 from *Cucumis sativus* (CDS dataset of the GenBank project PRJNA80169 v. 2^42^).

The nuclear contigs obtained from our skimming data (see Methods section 2) were also surveyed to identify putatively single copy nuclear loci that would be conserved enough to be aligned across Cucurbitaceae but with at least part of them variable enough to resolve relationships at lower taxonomic levels, especially within Sicyeae. In order to avoid multiple copy regions and paralogy problems, nuclear contigs were aligned using BLAST+^46^ (blastn) against the CDS of the published genome of *Cucumis sativus* (GenBank project PRJNA80169 v. 2^42^), and against themselves, and genes with more than one hit in a genomic dataset or in the genome of *C. sativus* were discarded. Genes mapping to organelle genomes were also discarded since they could represent paralogous copies of organelle genes that were transferred in the nuclear genome. This resulted in a set of 737 single-copy genes, of which we kept only the 143 that were present in all sampled species. A final check was performed to minimize the risk of paralogy by selecting only the genes for which all species would have their best hit against a same accession when blasted against the GenBank nucleotide database [https://blast.ncbi.nlm.nih.gov/Blast.cgi Accessed on 23 September 2016 with default parameters]. This resulted in 54 final genes, which were named based on their annotation in the published genome of *C. sativus*, and aligned to the *C. sativus* reference using MAFFT v. 7^47^ to identify exon/intron borders, revealing 14 genes with one intron and eight with two introns. Finally, the nuclear ribosomal RNAs 18S–5.8S–26 S and the ITS1 and ITS2 regions separating them were also recovered for each species, by finding the contig with the best blast hit to the nuclear RNA of *Trichosanthes kirilowii* (Genbank accession KM051446).

### Sequence alignments, phylogenetic inferences, and PI analyses

Coding and non-coding regions were extracted from all plastomes and aligned separately with MAFFT^47^ using the “global pair” approach for coding and the “genafpair” approach for non-coding regions, and 1000 iterations, resulting in 76 CDS, 87 NCS, and 20 intronic matrices. The same was done for the nuclear regions (including the ones obtained by Sanger sequencing), resulting in 57 CDS, 3 RNA, 2 ITS and 32 intronic matrices. Matrices were trimmed of their columns containing more than 70% gaps. All single regions were submitted to maximum likelihood phylogenetic analysis (ML) using RAxML v. 8.2.4^27^ with the GTRGAMMA model and 100 bootstrap replicates. They were also concatenated and submitted to the same ML analysis but with 1000 bootstrap replicates. Analyses were conducted with and without partitioning by locus. For *Bryonia* and *Momordica*, a different species was used to perform genome skimming and to sequence the regions “1_All_NEW”, “2_All_NEW” and “3_All_NEW”, so we concatenated the sequences of both species to build a representative sequence of the genus. We did the same for *Cucumis*, for which nuclear and plastid genes were also obtained from two different published datasets obtained from two species (see Supplementary Table S1). These genera are indicated as “*Genus* spp.” in the figures. Species trees were also inferred by summarizing the unrooted locus tree topologies with ASTRAL-III^28^, after collapsing nodes with less than 10% BS, as recommended in the software documentation. Species trees were rooted on *Corynocarpus laevigatus* using phyx^48^.

Phylogenetic informativeness for each locus was analysed with PhyDesign^49^. Following the recommendations of Townsend (http://phydesign.townsend.yale.edu/instructions.html) to use a “fairly well” resolved topology, PI estimations were performed with and without six so-called “conflicting taxa” that had different phylogenetic placements in different analyses, low locus support, and/or that were involved in a nucleo-cytoplasmic conflict (see Results section 2), namely *Bryonia*, *Luffa*, *Herpetospermum*, *Hodgsonia*, *Indofevillea*, and *Trichosanthes truncata*. To characterize locus PI through time, we delimited epochs of the evolution of Cucurbitaceae, so that one epoch would be circumscribed by two well-resolved divergences but contain divergences involving the conflicting taxa. Four epochs (e1 to e4) were defined as follows: e1: Present-(*T. kirilowii* + *T. tubiflora*); e2: (*T. kirilowii* + *T. tubiflora*)-*Nothoalsomitra*; e3: *Nothoalsomitra*- *Ampelosicyos*; e4: root-*Ampelosicyos*. Phydesign instructions^49^ recommend using HyPhy^50^ instead of DNArates^51^ to estimate locus rates but HyPhy could not run on some nuclear loci, so we used DNArates after controlling that rates estimated by both programs were almost identical (plot available on demand). Rate estimations were based on the concatenated matrix and the corresponding RAxML species tree, which was first made ultrametric in R^52^ using the chronos function of the package ape v. 3^53^, with root calibration of 1 and lambda = 0. The same analysis was performed on the matrix and trees without problematic taxa. An integrated PI (iPI) was calculated for each locus and each of the four epochs. To take into account that recent phylogenetic signal may have obscured past signal, a penalized integrated PI (iPI_pen_) was also calculated for each locus at each epoch in the following way: with time t increasing from the tips to the root of the tree, and with t_e_ being the medium time point of the considered epoch and t_m_ the point in time where PI was the highest across all epochs, if t_e_ < t_m_ (i.e. if the considered epoch was more recent than the time of maximal PI), iPI_pen_ = iPI, but if t_e_ > t_m_ (i.e. if the considered epoch was older than the time of maximal PI), iPI_pen_ = iPI*(PI at t_e_/PI at t_m_). We then classified each locus as misleading for a given epoch among e2, e3 and e4 if iPI_pen_ < iPI for that epoch, or non-misleading if iPI_pen_ = iPI for that epoch. Taxa sequences were then removed from a locus alignment if that locus was considered misleading for the epoch in which the taxon diverged from its sister taxon. Alignments and phylogenetic analyses were repeated with the filtered locus matrices.

Locus signal supporting each clade in the RAxML and ASTRAL species trees was analysed using phyparts^9^, which required locus trees to be rooted. Rooting was done with phyx^48^, using *Corynocarpus laevigatus* as an outgroup for all plastid loci and for all nuclear locus trees that included this taxon. For nuclear loci with missing taxa, loci were rooted on the most phylogenetically distant relative of *Trichosanthes* available. When phylogenetic uncertainty prevented outgroup assignment, the locus was discarded. Support analyses were repeated after collapsing all locus tree nodes with less than 70% BS. Trees, bar plots and box plots were performed with R^52^, using the packages ape v. 3^53^, cowplot (https://github.com/wilkelab/cowplot), ggimage (https://github.com/GuangchuangYu/ggimage), gplot^54^, ggtree^55^ and phytools^56^. Figures were manually edited in CorelDRAW 2019.

## Supplementary information

Supplementary materials.

## Data Availability

Raw Illumina reads have been submitted to the ncbi SRA under project number PRJNA566101 and fully assembled and annotated plastomes have been deposited in GenBank (see accession numbers in Supplementary Table S5).
