# Supplementary material for: Phylogenetic informativeness analyses to clarify past diversification processes in Cucurbitaceae
Source: Sci Rep. 2020 Jan 16;10:488. doi: 10.1038/s41598-019-57249-2 (PMC6965171; doi:10.1038/s41598-019-57249-2)
Supplement: Supplementary file 1 — Supplementary materials. [file 41598_2019_57249_MOESM1_ESM.pdf]

Phylogenetic informativeness analyses to clarify past diversification processes in Cucurbitaceae

Sidonie Bellot[1], Thomas C. Mitchell[2] & Hanno Schaefer[2]*

[1]Royal Botanic Gardens, Kew, TW9 3DS, Richmond, UK

[2]Plant Biodiversity Research, Department Ecology & Ecosystem Management, Technical University of Munich, Emil-Ramann Strasse 2, 85354 Freising, Germany

*corresponding author: hanno.schaefer@tum.de

Supplementary Material

Figure S1. Phylogenies of Cucurbitaceae based on all plastid and nuclear loci. a. ASTRAL multispecies coalescent phylogeny inferred by summarizing all nuclear and plastid locus trees. Pie charts above branches represent the percentage of loci that agree (blue), disagree (one of the main alternatives: green, other alternatives: red), or are neutral (grey) with respect to the clade descending from the branch. Pie charts below branches represent the same but after collapsing nodes with BS < 70% in locus trees. Node labels are arbitrary numbers for easier description in the text. See Results section 2 for details. **b.** RAxML maximum likelihood (ML) tree inferred from a concatenated alignment of all loci. Numbers next to the nodes are the percentages of bootstrap replicates supporting the nodes.

Figure S2. Penalized and original phylogenetic informativeness of all nuclear and plastid loci, integrated over each epoch, with (a) or without (b) including conflicting taxa. See Methods section 4 for details.

Figure S3. Phylogenetic informativeness of all nuclear and plastid loci, integrated over each epoch, with (a) or without (b) conflicting taxa. Loci classified as "1-2-3-4" were predicted to be non-misleading in all epochs, loci classified as "1-2-3" were predicted to be misleading for divergence events that happened in epoch 4, and loci classified as "1-2" were predicted to be misleading for divergence events that happened in epochs 3 or 4. See Methods section 4 and Results section 3 for details.

Figure S4. Phylogenies of Cucurbitaceae obtained by analysing all plastid and nuclear

loci after data filtering. a. Phylogeny obtained by coalescence summary (using ASTRAL) of all ML plastid and nuclear locus trees. **b.** Phylogeny obtained by coalescence summary (using ASTRAL) of all ML nuclear locus trees and of a single plastid tree obtained by ML analysis of a concatenated alignment of all plastid loci. Pie charts above branches represent the percentage of loci that agree (blue), disagree (one of the main alternatives: green, other alternatives: red), or are neutral (grey) with respect to the clade descending from the branch. Pie charts below branches represent the same but after collapsing nodes with BS < 70% in locus trees. Node labels are arbitrary numbers for easier description in the text. Inset: bar plot representing locus support for node 15. See Results section 2 and Methods section 4 for details.

Table S5. Sampled species with voucher and data availability information.

Figure S1

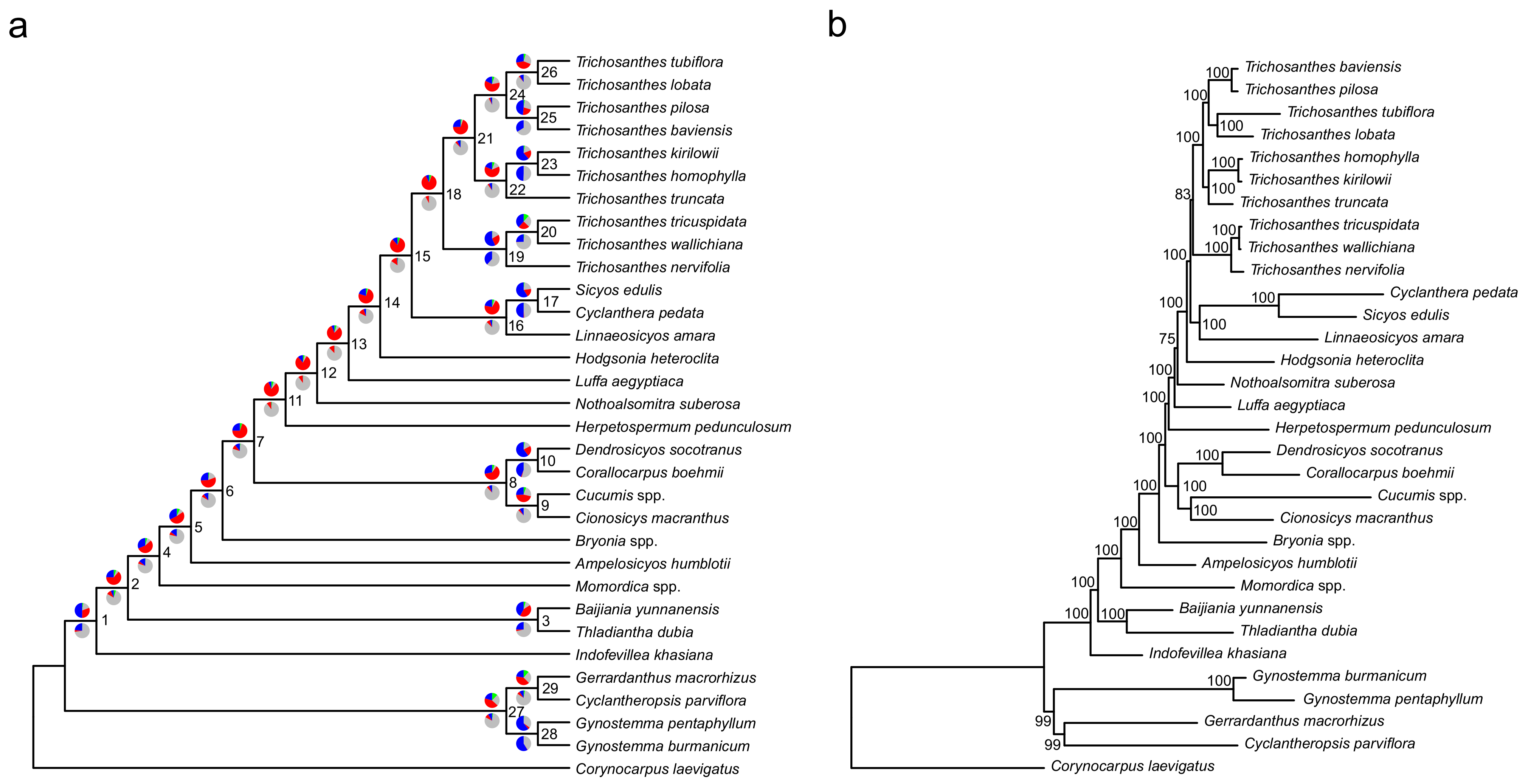
a
b
Trichosanthes tubiflora
Trichosanthes lobata
Trichosanthes pilosa
Trichosanthes baviensis
Trichosanthes kirilowii
Trichosanthes homophylla
Trichosanthes truncata
Trichosanthes tricuspidata
Trichosanthes wallichiana
Trichosanthes nervifolia
Sicyos edulis
Cyclanthera pedata
Linnaeosicyos amara
Hodgsonia heteroclita
Luffa aegyptiaca
Nothoalsomitra suberosa
Herpetospermum pedunculosum
Dendrosicyos socotranus
Corallocarpus boehmii
Cucumis spp.
Cionosicys macranthus
Bryonia spp.
Ampelosicyos humblotii
Momordica spp.
Baijiania yunnanensis
Thladiantha dubia
Indofevillea khasiana
Gerrardanthus macrorhizus
Cyclantheropsis parviflora
Gynostemma pentaphyllum
Gynostemma burmanicum
Corynocarpus laevigatus

Figure S2

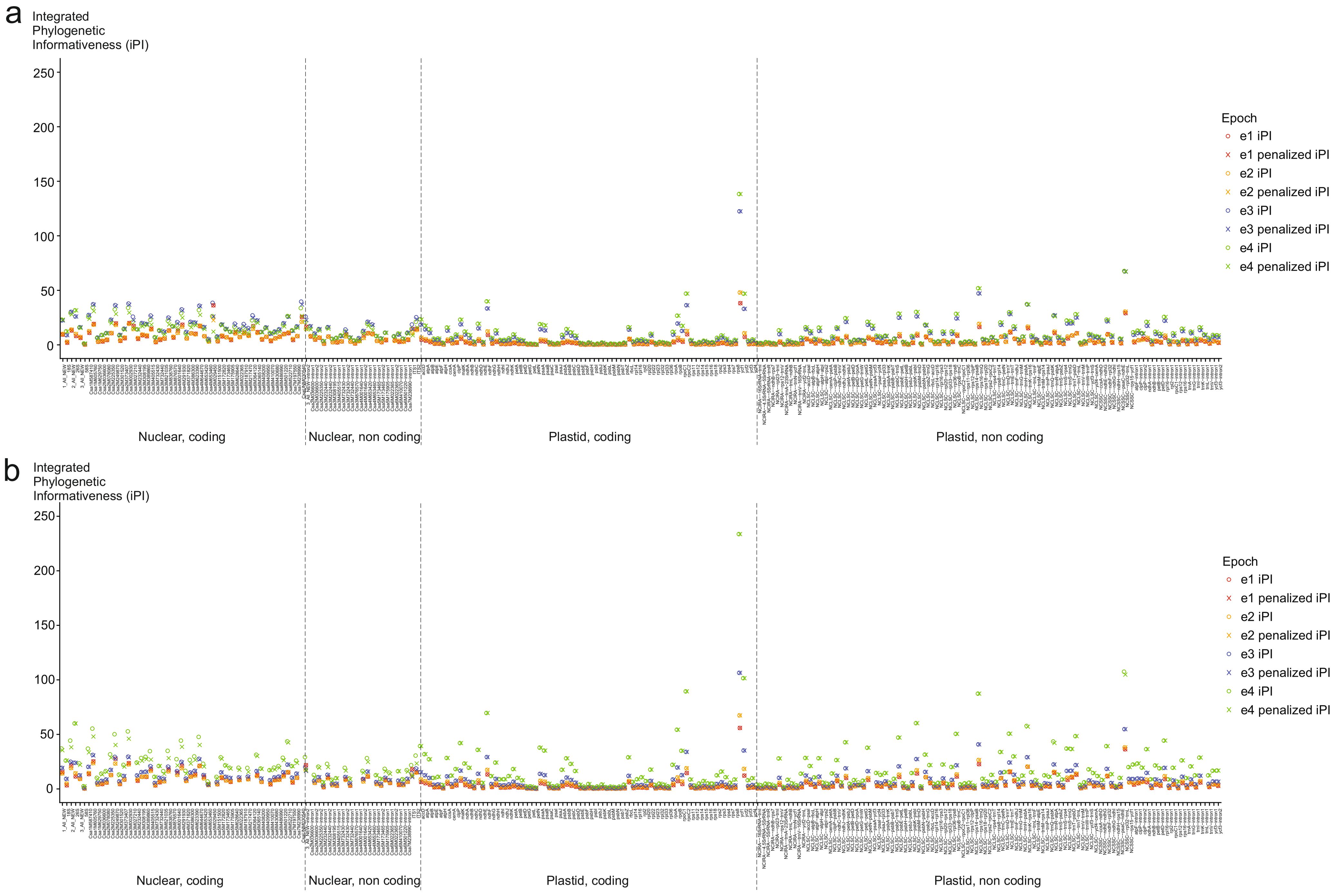
a
Integrated
Phylogenetic
Informativeness (iPI)
0
50
100
150
200
250
Nuclear, coding
Nuclear, non coding
Plastid, coding
Plastid, non coding
Epoch
e1 iPI
e1 penalized iPI
e2 iPI
e2 penalized iPI
e3 iPI
e3 penalized iPI
e4 iPI
e4 penalized iPI
b
Integrated
Phylogenetic
Informativeness (iPI)
0
50
100
150
200
250
Nuclear, coding
Nuclear, non coding
Plastid, coding
Plastid, non coding
Epoch
e1 iPI
e1 penalized iPI
e2 iPI
e2 penalized iPI
e3 iPI
e3 penalized iPI
e4 iPI
e4 penalized iPI

Figure S3

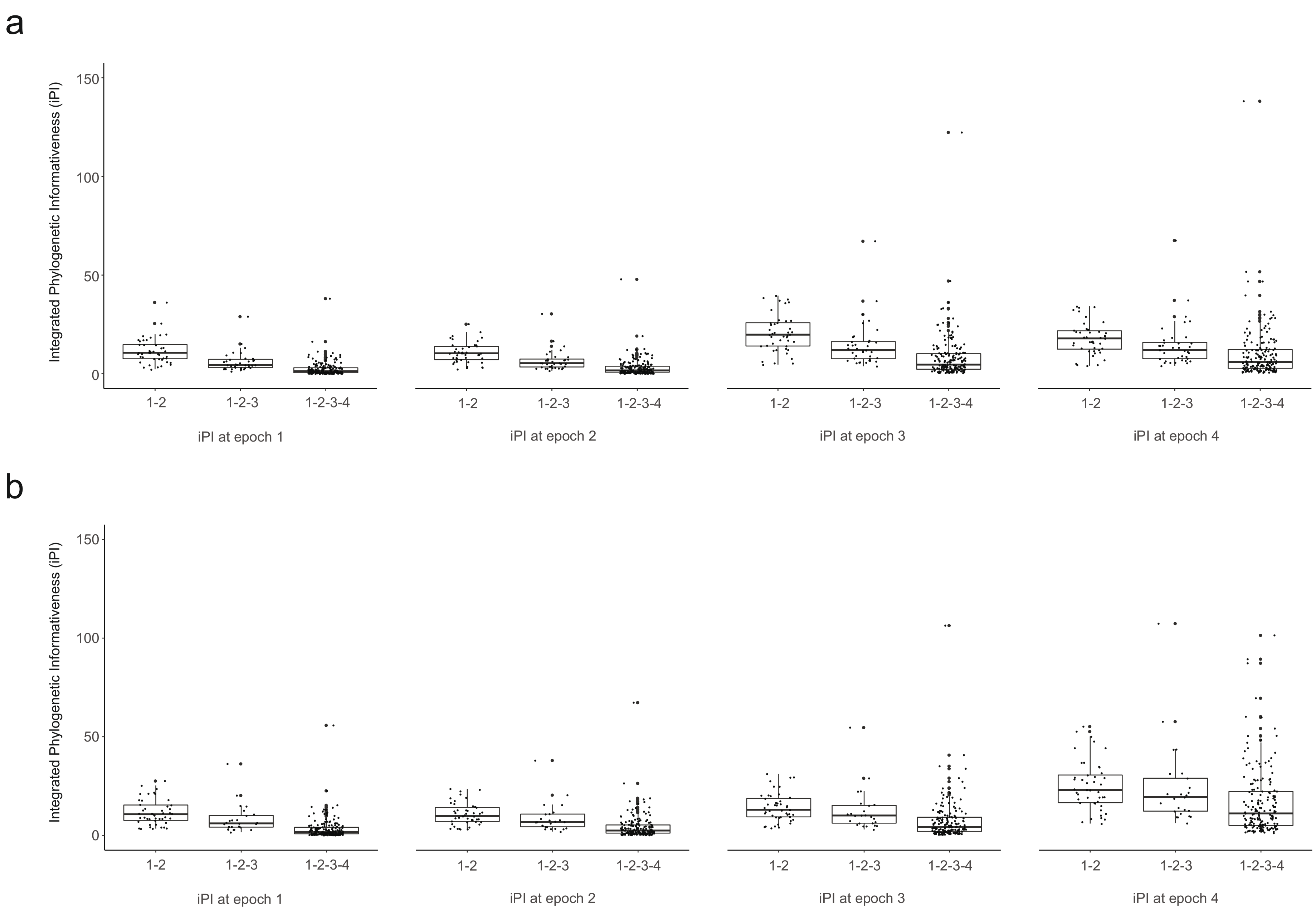
a
Integrated Phylogenetic Informativeness (iPI)
0
50
100
150
1-2
1-2-3
1-2-3-4
iPI at epoch 1
iPI at epoch 2
iPI at epoch 3
iPI at epoch 4
b
Integrated Phylogenetic Informativeness (iPI)
0
50
100
150
1-2
1-2-3
1-2-3-4
iPI at epoch 1
iPI at epoch 2
iPI at epoch 3
iPI at epoch 4

Figure S4

a

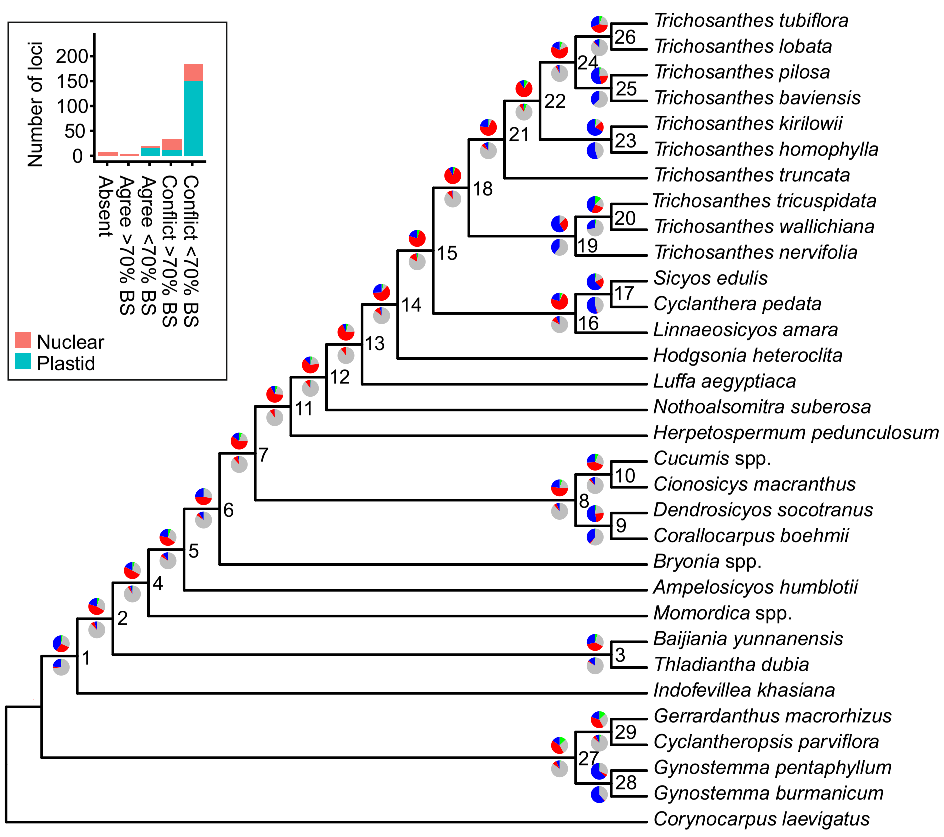

Number of loci
200
150
100
50
0
Absent
Agree >70% BS
Agree <70% BS
Conflict >70% BS
Conflict <70% BS
Nuclear
Plastid
Trichosanthes tubiflora
Trichosanthes lobata
Trichosanthes pilosa
Trichosanthes baviensis
Trichosanthes kirilowii
Trichosanthes homophylla
Trichosanthes truncata
Trichosanthes tricuspidata
Trichosanthes wallichiana
Trichosanthes nervifolia
Sicyos edulis
Cyclanthera pedata
Linnaeosicyos amara
Hodgsonia heteroclita
Luffa aegyptiaca
Nothoalsomitra suberosa
Herpetospermum pedunculosum
Cucumis spp.
Cionosicys macranthus
Dendrosicyos socotranus
Corallocarpus boehmii
Bryonia spp.
Ampelosicyos humblotii
Momordica spp.
Baijiania yunnanensis
Thladiantha dubia
Indofevillea khasiana
Gerrardanthus macrorhizus
Cyclantheropsis parviflora
Gynostemma pentaphyllum
Gynostemma burmanicum
Corynocarpus laevigatus

b

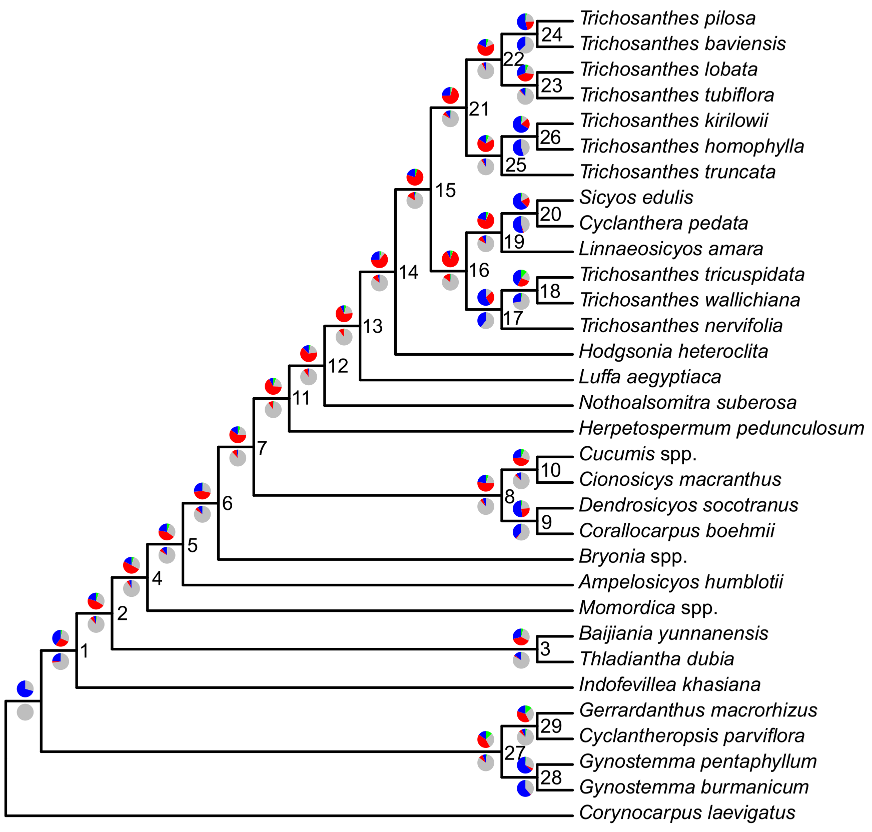

Trichosanthes pilosa
Trichosanthes baviensis
Trichosanthes lobata
Trichosanthes tubiflora
Trichosanthes kirilowii
Trichosanthes homophylla
Trichosanthes truncata
Sicyos edulis
Cyclanthera pedata
Linnaeosicyos amara
Trichosanthes tricuspidata
Trichosanthes wallichiana
Trichosanthes nervifolia
Hodgsonia heteroclita
Luffa aegyptiaca
Nothoalsomitra suberosa
Herpetospermum pedunculosum
Cucumis spp.
Cionosicys macranthus
Dendrosicyos socotranus
Corallocarpus boehmii
Bryonia spp.
Ampelosicyos humblotii
Momordica spp.
Baijiania yunnanensis
Thladiantha dubia
Indofevillea khasiana
Gerrardanthus macrorhizus
Cyclantheropsis parviflora
Gynostemma pentaphyllum
Gynostemma burmanicum
Corynocarpus laevigatus

Table S5. Table of material with voucher list and data availability information.

Species	Voucher information	accession numbers (Illumina reads)	accession numbers (plastome)
Ampelosicyos humblotii (Cogn.) Jum. & H. Perrier	Mitchell & Schaefer 19 (TUM)	SRR10137796	MN542396
Baijiania yunnanensis (A.M.Lu & Z.Y.Zhang) A.M.Lu & J.Q.Li	H. Schaefer HSC 73 (TUM)	SRR10137784	MN542405
Bryonia marmorata E. Petit	H. Schaefer 2015/087 (TUM) (Genome skimming)	SRR10137795	MN542386
Cionosicys macranthus (Pittier) C.Jeffrey	H. Schaefer HSC 60 (TUM)	SRR10137773	MN542397
Corallocarpus boehmii (Cogn.) C. Jeffrey	H. Schaefer 2014/320 (TUM)	SRR10137774	MN542408
Cyclanthera pedata (L.) Schrad.	S.S. Renner, R. Omlor, G. Hausner 2805 (TUM)	SRR10137771	MN542391
Cyclantheropsis parviflora (Cogn.) Harms	H. Schaefer 2013/088 (TUM)	SRR10137772	MN542399
Dendrosicyos socotranus Balf.f.	H. Schaefer 2015/091 (TUM)	SRR10137770	MN542400
Gerrardanthus macrorhizus (Harv.) ex. Benth. & Hook.f.	H. Schaefer 2014/324 (TUM)	SRR10137768	MN542407
Gynostemma burmanicum King ex. Chakrav.	H. Schaefer 2014/326 (TUM)	SRR10137769	MN542403
Herpetospermum pedunculosum (Ser.) C.B.Clarke	H. Schaefer 2015/040 (TUM)	SRR10137793	MN542388
Hodgsonia heteroclita (Roxb.) Hook.f. & Thomson	H. Schaefer, D.A. Santamaria & D.E. Boufford 071 (MAND)	SRR10137794	MN542387
Indofevillea khasiana Chatterjee	H. Schaefer, D.A. Santamaria & D.E. Boufford 154 (MAND)	SRR10137792	MN542389
Linnaeosicyos amara (L.) H.Schaef. & Kocyan	T.C. Mitchell 003 (TUM)	SRR10137790	MN542401
Luffa aegyptiaca Mill.	H. Schaefer, D.A. Santamaria & D.E. Boufford 019 (MAND)	SRR10137791	MN542383
Momordica sessilifolia Cogn.	H. Schaefer 2019/023 (TUM)	SRR10137789	MN542406
Nothoalsomitra suberosa (F.M.Bailey) I.Telford	I. R. Telford 12487 (NE)	SRR10137788	MN542410
Sicyos edulis L.	H. Schaefer 2012/536 (TUM)	SRR10137787	MN542384
Thladiantha dubia Bunge	S.S. Renner, R. Omlor, G. Hausner 2780 (TUM)	SRR10137785	MN542382
Trichosanthes baviensis Gagnep.	H. Schaefer, D.A. Santamaria & D.E. Boufford 447 & 459 (MAND)	SRR10137786	MN542385
Trichosanthes homophylla Hayata	H. Schaefer HSC 33 (TUM)	SRR10137782	MN542394
Trichosanthes kirilowii Maxim.	H. Schaefer 2019/025 (TUM)	SRR10137781	MN542395
Trichosanthes lobata Roxb.	H. Schaefer 2019/026 (TUM)	SRR10137780	MN542404
Trichosanthes nervifolia L.	H. Schaefer HSC 67 (TUM)	SRR10137779	MN542398
Trichosanthes pilosa Lour.	H. Schaefer, D.A. Santamaria & D.E. Boufford 374 (MAND)	SRR10137778	MN542402
Trichosanthes tricuspidata Lour.	H. Schaefer, D.A. Santamaria & D.E. Boufford 336 (MAND)	SRR10137783	MN542392
Trichosanthes truncata C.B. Clarke	H. Schaefer, D.A. Santamaria & D.E. Boufford 072 (MAND)	SRR10137777	MN542409
Trichosanthes tubiflora (Wight & Arn.) H.J. de Boer	H. Schaefer s.n. (TUM)	SRR10137776	MN542393
Trichosanthes wallichiana (Ser.) Wight	H. Schaefer 2015/095 (TUM)	SRR10137775	MN542390
Corynocarpus laevigatus J.R. Forst. & G. Forst.	GenBank	-	NC014807
Cucumis hystrix Chakrav.	GenBank	-	NC023544
Cucumis sativus L.	GenBank	-	PRJNA33619 (genome)
Gynostemma pentaphyllum (Thunb.) Makino	GenBank	-	NC029484
Luffa sp.	GenBank	-	PRJNA225972 (transcriptome)
Momordica charantia L.	GenBank	-	PRJNA213805 (transcriptome)
Siraitia grosvenorii (Swingle) C. Jeffrey ex A.M. Lu & Z.Y. Zhang	GenBank	-	PRJNA67121 (transcriptome)
